# The Properties of the Monolayers of Sorbitan Lipids as Informative Factors on the Hydrophilic–Lipophilic Balance Value of Their Mixtures, Proposed for Dermatological Applications

**DOI:** 10.3390/molecules30081841

**Published:** 2025-04-19

**Authors:** Remigiusz Zapolski, Monika Gasztych, Beata Jastrząb-Miśkiewicz, Alina Jankowska-Konsur, Witold Musiał

**Affiliations:** 1 Department of Physical Chemistry and Biophysics, Pharmaceutical Faculty, Wroclaw Medical University, Borowska 211, 50-556 Wroclaw, Poland; remigiusz.zapolski@gmail.com (R.Z.); monika.gasztych@umw.edu.pl (M.G.); 2 University Centre for General and Oncological Dermatology, Wroclaw Medical University, Borowska 213, 50-556 Wroclaw, Poland; bajastrzab@gmail.com (B.J.-M.); alina.jankowska-konsur@umw.edu.pl (A.J.-K.)

**Keywords:** surfactant, HLB, π–A isotherms, surface pressure measurements

## Abstract

This study investigates the correlation between the hydrophilic–lipophilic balance (HLB) values and the π–A isotherm parameters of surfactant monolayers composed of sorbitan esters, specifically sorbitan monopalmitate, sorbitan tristearate, sorbitan monooleate, and sorbitan sesquioleate. The surfactant mixtures were prepared, and their π-A isotherms were recorded. The HLB values calculated for each mixture were in the range 2.10–6.70. The HLB values were compared to compression parameters, including the ratios of the slopes and the ratios of the intercepts, which were between 0.19 and 4.00 and between 0.64 and 1.77, respectively, across the monolayer compression stages. The findings indicate a significant relationship between HLB values and isotherm parameters, particularly for systems with sorbitan monooleate and sorbitan sesquioleate. A value of determination coefficient of 0.95 was found for the linear equation representing the slope ratios as a function of HLB, whereas the intercept ratios, as a linear function of HLB, gave a lower value of 0.71. The results allow for the use of the π–A Langmuir isotherm to experimentally estimate the value of the HLB in mixtures of the sorbitol esters of fatty acids, whose value is an important parameter in the selection of optimized topical and transdermal formulations, highlighting the specific formulations that enhance active substance delivery while minimizing skin irritation potential.

## 1. Introduction

The effective delivery of active substances into and through the skin is crucial for various topical, transdermal, dermatological, and personal care applications. Although small, lipophilic molecules can traverse the skin with relative ease, the penetration of larger, hydrophilic molecules is significantly impeded by the stratum corneum’s low permeability [1,2,3].

Chemical agents such as surfactants, fatty acids, esters, and amines can be employed to enhance penetration. These substances infiltrate the skin and disrupt the structural organization and packing of the lipids and proteins within the stratum corneum [1,4]. While chemical penetration enhancers effectively increase skin permeability for certain molecules, their use may be associated with irritation, limiting their applicability in practical scenarios [5,6].

**Figure 1:**
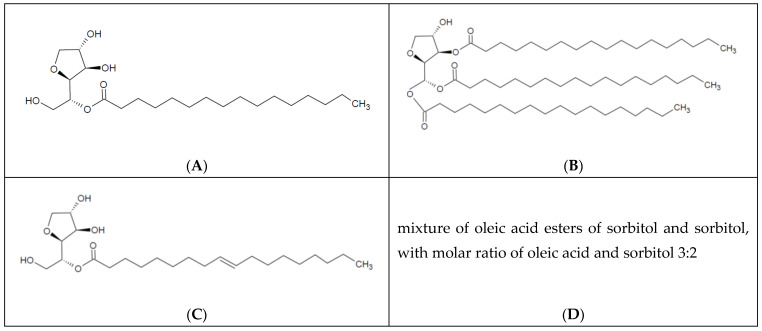
Schematic chemical structure of evaluated spans: (**A**) sorbitan monopalmitate (S40), (**B**) sorbitan tristearate (S65), (**C**) sorbitan monooleate (S80), and (**D**) sorbitan sesquioleate (S83).

Among these surfactants, the sorbitan esters from the so-called Span group, specifically sorbitan monopalmitate, sorbitan tristearate, sorbitan monooleate, and sorbitan sesquioleate, have garnered significant attention for their multifaceted applications in topical formulations [4,5]. The basic structural characterization of the molecules is presented below in Figure 1.

Sorbitan monooleate is widely utilized in the formulation of oil-in-water emulsions, where it facilitates the solubilization of lipophilic drugs while improving the sensory attributes of creams and lotions [7]. Importantly, a recent study reported on cubosomes and hexosomes stabilized by sorbitan monooleate as a biocompatible nanoplatform for targeting skin metastatic human melanoma [8]. Similarly, sorbitan monopalmitate has been shown to enhance moisture retention in various topical applications, proving beneficial in formulations designed for dry or sensitive skin [7]. Furthermore, the study conducted by Abdelkader et al. focused on the formulation and optimization of lipid- and poloxamer-tagged niosomes for the dermal delivery of terbinafine. In this study, different lipids/surfactants with two different molar ratios were investigated with sorbitan monopalmitate-based niosomes. Both alternatives were identified as potential substitutes for cholesterol as bilayer membrane stabilizers for terbinafine, demonstrating superior antifungal activities in vitro, as indicated by the inhibition zones against Candida albicans ATCC 60913 [9]. Sorbitan tristearate and sorbitan sesquioleate are particularly effective in stabilizing emulsions and improving the delivery of active substances, making them suitable for formulations targeting dermatological conditions such as eczema [5,7,10]. Despite their benefits, the safety profiles of Span surfactants must be meticulously evaluated to mitigate potential adverse effects. While generally considered safe for topical use, some studies report skin irritation or sensitization, particularly at higher concentrations. For example, sorbitan sesquioleate, commonly incorporated in various highly potent to super-potent corticosteroids, has been reported as an important contact allergen [11,12]. Therefore, careful formulation practices and thorough safety assessments are paramount to ensure the well-being of consumers using these surfactants.

A hydrophilic–lipophilic balance (HLB) value plays an important role in the prediction of the properties of a formulation, as well as being beneficial to production procedures. Depending on the substance which should be applied topically on the skin surface, various formulations may be developed to ensure proper or modified drug release and transport through the skin. The HLB level of the surfactant added to the biphasic or multicomponent mixture may enable the formation of creams or ointments with an internal structure governed by interfacial phenomena. The HLB value influences, i.a., the type of emulsion formed, o/w or w/o; in some cases, multiple emulsions may emerge. The programmed HLB value of some formulation components influences the solubility of the active pharmaceutical ingredient. The HLB scale is stretched between ca. 1, with antifoaming agents at the minimal edge, and ca. 20, with solubilizing agents [13,14,15]. The surfactants and their mixtures with HLB values of 3–6 have a potential to stabilize w/o emulsions, whereas the higher values between 8 and 16 usually favor the systems with o/w emulsions. Between the surfactants applied for the stabilization of the emulsions w/o there are nonionic surface-active components based on sorbitan and long chain fatty acids [16,17,18,19]. Within this group sorbitan monopalmitate, sodium tristearate, sodium monooleate, as well as sodium sesquioleate may be applied to formulate stable systems with a continuous lipid phase or as cofactors in other types of formulation for controlled drug delivery [20,21,22,23]. The basic properties of this group are gathered in Table 1.

An evaluation of an HLB value encounters numerous challenges. An HLB value can be interpreted as the fraction of hydrophilic functional groups compared to lipophilic functional groups, presented as a single number. However, determination of this value can be performed by both experimental and computational methods. Surface activity is a factor that is strongly related to the amphiphilic properties of molecules and can be related to the HLB value. In our former work, we investigated the possibility of excavating the HLB of surfactants systems from the data obtained in experiments on the monolayers of surfactants, using algorithms from the response surface methodology, and on the mixtures of sorbitan derivatives, with sorbitan monopalmitate as the main component, modified by the addition of other sorbitan esters [24]. The evaluation of dependency surface pressure–surface area was proposed by Langmuir and developed by numerous researchers [25]. On the other hand, the well-known parameters ascribed to the course of isotherms are the compression factor (*E*) or compression modulus, calculated as a ratio of surface pressure change (∆π) to area change (*∆S*) at a certain surface area (*A*). The plot of E against S may be informative in terms of the mechanical properties of the monolayer [26].

(1) $$E=-A{\left(\frac{∆\pi }{∆A}\right)}_{T}$$

The elastic properties of monolayers are presented in varied forms, including the reciprocal compression modulus, compressibility (*C*) [27].

(2) $$C=\frac{1}{E}=-\frac{1}{A}{\left(\frac{∆A}{∆\pi }\right)}_{T}$$

However, the data are derived directly from the slopes of the graphs, which represent the change of the ∆π with the decreasing surface of the monolayer, as it was expressed by Vollhardt and Fainerman, so the basic approach is anchored in the slope of the linear surface pressure–surface area relationship [28].

The aim of this study was to search for distinct parameters of π-A isotherms that can reflect the HLB value in surfactant mixtures prepared based on sorbitan sesquioleate with other surfactants forming a sorbitan ester group. The specific aim was to evaluate the π-A isotherms of surfactant mixtures and to identify the relationship between the slopes or interceptions of the linear segments of π-A isotherms and the HLB values of the mixtures. The potential relationship could be used, after further validation, in a pioneering HLB quantification from a direct isotherm evaluation.

## 2. Results

### 2.1. Recorded Surface Pressure of Evaluated Monolayers

**Figure 2:**
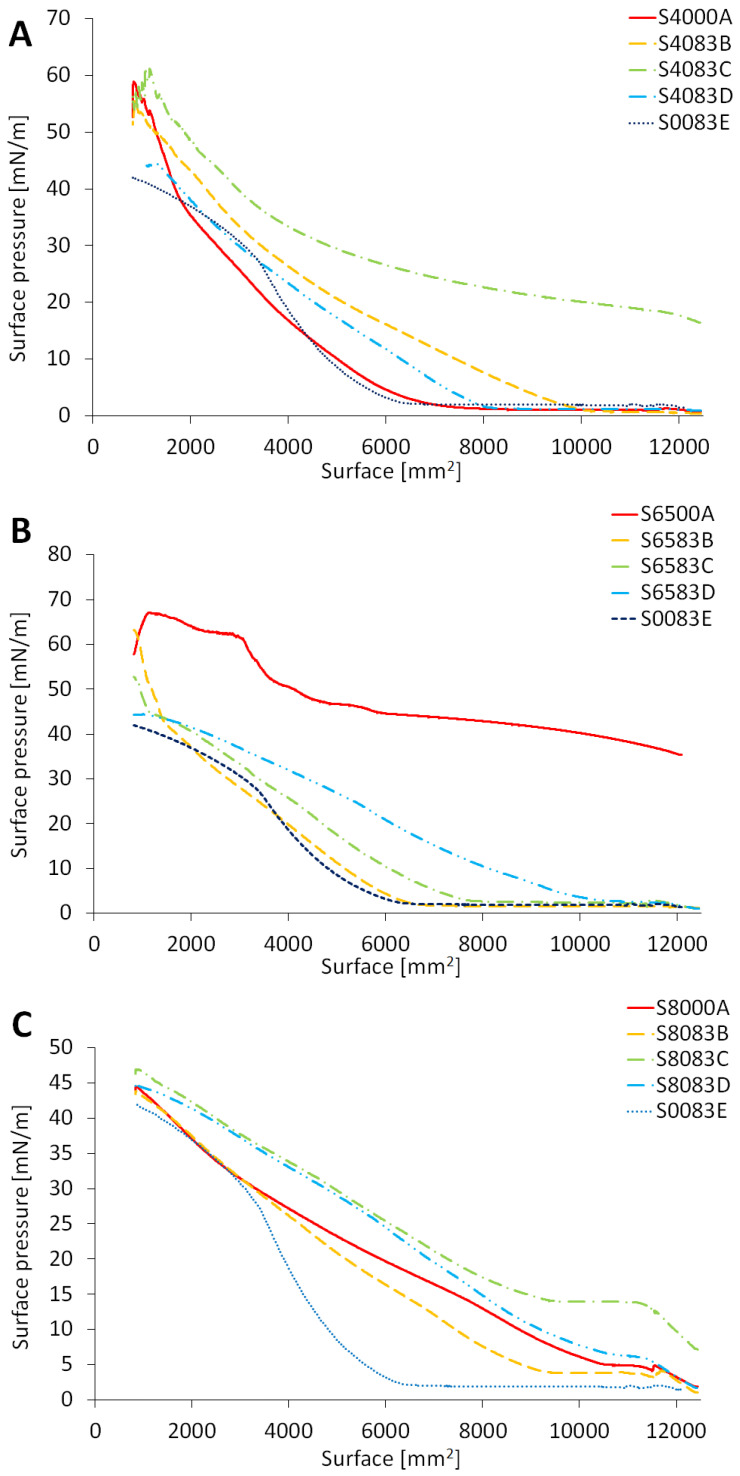
Exemplification of isotherms of evaluated sorbitan esters and the mixtures: S40:S83 (**A**), S65:S83 (**B**), and S80:S83 (**C**); the composition and abbreviations of presented isotherms are given in Table 5 in Section 4.

The surface pressure was measured in the range of a surface area of ca. 900–12,000 mm^2^. The gathered data recorded for the monolayers containing various proportions of sorbitan monopalmitate and sorbitan sesquioleate were in the range between 0.9 and 60.9 mN/m, with the highest values through the entire process of compression recorded for the system which contained the surfactants in the proportion 0.72:0.28, respectively (Figure 2A, S4083D).

The monolayers composed of a mixture of sorbitan tristearate and sorbitan sesquioleate were characterized by surface pressures between 0.9 and 67.0 mN/m: however, the highest localization of an isotherm on the graph was recorded in the case of the monolayer composed of sorbitan tristearate only (Figure 2B, S6500A). The above-mentioned systems had the plots of a shape close to the often-recognized pattern of the so-called π-A isotherm, and characteristic inflection points were observed on part of the considered plots (Figure 2A,B). The course of the relationship between surface pressure and the monolayer surface of systems with sorbitan monooleate and sorbitan sesquioleate was shaped differently in comparison to the formerly presented case, with long parts of almost straight-line plots (Figure 2C).

As mentioned above, the sorbitan sesquioleate is a mixture of sorbitan monooleate and oleic acid. Two molecules of sorbitan monooleate are accompanied by one molecule of oleic acid. Actually, the combination of S80 with S83 should be considered as dilution of sorbitan monooleate by oleic acid; one added mole of sorbitan sesquiolete adds 1/3 mole of oleic acid, and 2/3 moles of sorbitan monoleate. The dilution is not very intensive, thus the plots on Figure 2C are shaped differently compared to Figure 2A,B. From Figure 2C and the proportions of S80 and S83, it may be concluded that the plots variability is less expressed, as in the case of S83 added to S40 or S65. The plots on Figure 2C are close to each other (cf. the scale on the y-axis of Figure 2C).

The range for the surface pressure was 1.1–46.6 mN/m. The highest values for surface pressure were recorded for the system composed of the surfactants in the molar composition 0.72:0.28, as in the case of the system composed on the base of sorbitan monopalmitate (S8083C).

### 2.2. The Stages of Compression Identified by Linear Sections of the Plots

It was possible to distinguish on the plots the stages of the compression processes. The slopes, and the intercepts of the respective equations, which reflected the two main stages, were gathered in Table 2. The calculated slopes of the first stage of compression (a_1_) were lower in standard numbers, when compared to the slopes calculated for the second stage of compression (b_1_) for the preparations composed of sorbitan monopalmitate and doped by sorbitan sesquioleate (Table 2, S4000A-D). A similar tendency was observed for the intercepts of the y-axis; however, the monolayer composed of sorbitan sesquioleate only did not fit that tendency (Table 2, S0083E).

The maximal and minimal absolute values of the slopes for the preparations composed of sorbitan tristearate and doped with sorbitan sesquioleate in the first stage were 2.94 × 10^−2^ and 5.90 × 10^−3^, respectively. The second stage of compression resembled absolute values in the range of 4.00 × 10^−3^ to 5.19 × 10^−2^. The maximal and minimal interceptions represented the values of 127.42 and 51.78, respectively (Table 2, S6500A-D).

In the case of the monolayers prepared with the use of sorbitan monooleate and sorbitan sesquioleate (Table 2, S8000A-D), the absolute values of a_1_ were closer to each other between 4.10 × 10^−3^ and 4.40 × 10^−3^, compared to other types of mixtures. The absolute a_2_ values were between 2.40 × 10^−3^ and 6.60 × 10^−3^. The variability between the hypothetical first and second stages in the terms of intercepts (b_1_, b_2_) was less expressed, when compared to the preparations of sorbitan monopalmitate or sorbitan tristearate.

The determination coefficients for the selected linear sections of the graphs were in the ranges of 0.9914–0.9999 and 0.9731–0.9998, for the first and second stages of the process, respectively, as is presented in Table 2.

### 2.3. HLB Values of Evaluated Systems

In Table 3, we gathered the calculated values of the prepared surfactants mixtures, according to the values of HLB presented in the available bibliography. The highest levels of HLB were ascribed to the systems composed of sorbitan monopalmitate with sorbitan sesquioleate in the range between 4.25 and 5.62 (Table 3, S40:S83). The intermediate values were found for the systems comprising sorbitan monooleate and sorbitan sesquioletae, which were in the limits of 3.98–4.06 (Table 3, S80:S83). The lowest values were ascribed to the systems of sorbitan tristearate and sorbitan sesquioleate, i.e., 2.61–3.39 (Table 3, S65:S83). In the same table, we gathered the values of HLB for the systems with single surfactants, which were between 2.10 for the monolayer composed of sorbitan tristearate (S6500A) and 6.70 when the monolayer of sorbitan monopalmitate was considered (S4000A). The sorbitan monooleate and sorbitan sesquioleate were characterized by intermediate values of 4.30 and 3.70, respectively.

## 3. Discussion

### 3.1. Recorded Surface Pressure of Evaluated Monolayers

The recorded isotherms of the monolayers composed of sorbitan monopalmitate only (Figure 2A, S4000A), as well as the sorbitan sesquioleate only (Figure 2B, S0083E) monolayers pass at similar values on the common graph; however, in the case of sorbitan sesquioleate, we did not observe any characteristic curve break, which is often ascribed to the destruction of the monolayer. Unfortunately, the data from the binary monolayers did not have conclusive results (Figure 2A, S4083B-D). However, the presence of sorbitan monopalmitate ensures the appearance of a characteristic peak in the isotherms of monolayers also containing sorbitan sesquioleate, which was observed below 2000 mm^2^. It may be assumed that the sorbitan monopalmitate modified the structure of the monolayer to enable its breakage. In the case of pure sorbitan sesquioleate, the monolayer possibly possesses a high amount of spare space between the molecules, or its composition allows the layers to slide freely without breaking.

The apostasy from the standard shape of π-A in the case of S65500A sample is similar to the deviation of dioleoyl-sn-glycerophosphatidylcholine (DOPC) from dipalmitoyl-sn-glycerophosphatidylcholine (DPPC), observed by Anton et al. [32] during the study of a component of a pulmonary surfactant. In the case of S65 only, similar to the case of DOPC, the relatively huge nonpolar biforked chains may enable a systemic decrease in the monolayer surface area within a relatively stable surface pressure range. Oppositely, the DPPC was characterized by a decisive increase in surface pressure with the decrease in surface area. In the case of S65, the addition of a co-surfactant could diminish the range of stable surface pressure.

Doping sorbitan sesquioleate with a monolayer of sorbitan tristearate resulted in a very significant reduction of the surface pressure value (Figure 2B, S6583B). However, the further addition of sorbitan sesquioleate reversed the trend. The higher concentration of sorbitan sesquioleate moved the results (Figure 2B, S6583C,D) again towards the plot of the sorbitan tristearate monolayer (Figure 2B, S6500A). These phenomena confirm some interactions which could be further studied. The interactions between surfactants in a mixed monolayer are of high interest, and were studied, i.a., by Rosen and Zhou who assessed the βσ parameter, for which a negative value may confirm positive forces between the molecules of the surfactant [33]. Due to Lu et al., the interactions may be elucidated by relative molecular bulk based on different numbers of acyl chains and POE substituents in the molecules of surfactants [34]. One of the reasons for the observed variability of the S65:S83 system may be the low aqueous solubility of sorbitan tristearate [35,36].

The isotherms obtained from the measurements of the surface pressure with the decreasing area of the monolayers formed of sorbitan monooleate (Figure 2C, S8000A), as well as sorbitan monooleate and sorbitan sesquioleate (Figure 2C, S8083B-D), revealed elongated plots, with parallel sections, whereas the monolayer collapse was rather vague. As was mentioned above, the lack of a distinct breakage of the curve, which was reflected by the low slope ratio a_1_:a_2_ in Table 2, may be attributed to the high fluidity of the systems with sorbitan sesquioleate. The addition of sorbitan monooleate would not influence the course of the isotherm much, due to the structural similarities of sorbitan monooleate and sorbitan sesquioleate. In this study, we limited the experimental design to the tests available in some basic industrial laboratories focused on application research.

### 3.2. Calculated HLB

The determination of HLB values by calculative and analytical methods is an important task, with high consequences for the preparative and analytical procedures applied for the dermatological forms of drugs. The possibility of a formation of the required type of emulsions, as well as the solubilizing properties of surfactants, is strictly connected with the deciding influence of HLB. The hydrophilic–lipophilic balance of the assessed systems may be calculated according to the equations given by Griffin [37] and discussed in detail, i.a., by Pasquali et al. [38]. Table 4 covers the mostly accepted values of HLB of the assessed surfactants, and the resulting HLB of their mixtures, calculated on an arithmetic basis. As is clear from Table 3, the increasing fractions of sorbitan sesquioleate stimulated a decrease in the HLB in the mixtures with sorbitan monopalmitate and with sorbitan monooleate. Oppositely, when sorbitan sesquioleate was gradually added to sorbitan tristearate, the HLB increased.

### 3.3. The Stages of Compression Identified by Linear Sections of the Plots in the Context of HLB Values

**Figure 3:**
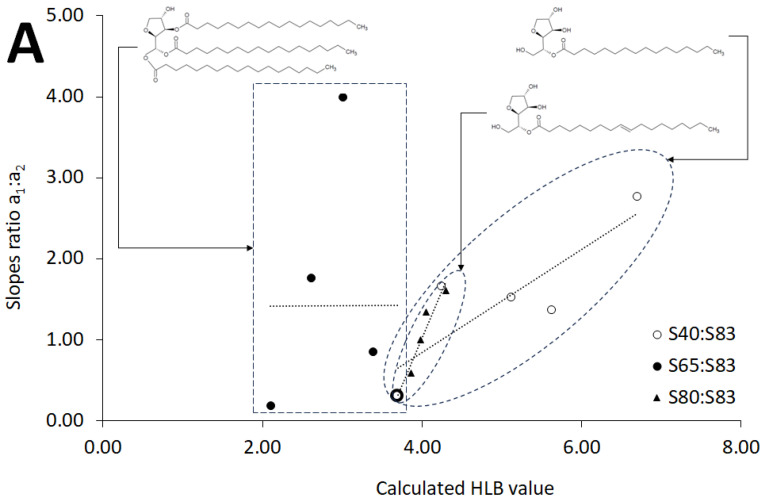

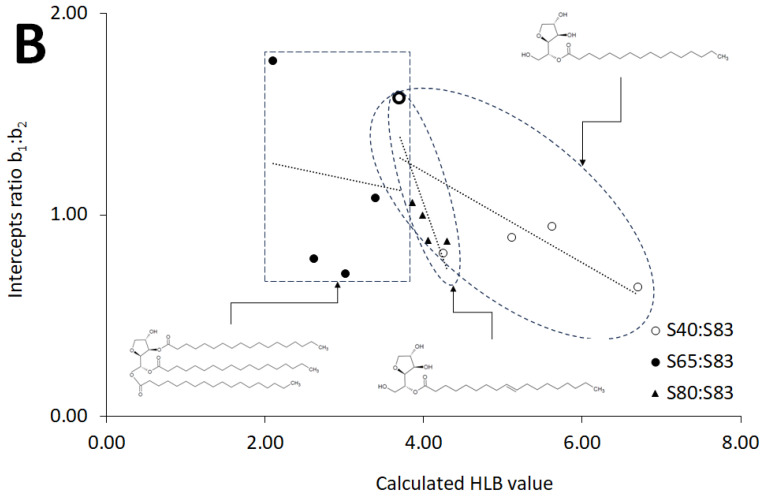
The influence of the calculated hydrophilic–lipophilic balance value (HLB) on (**A**) the ratio of the linear sections’ slopes in the first and second stages of compression (a_1_:a_2_) and on (**B**) the ratio of the linear sections’ y-intercepts (b_1_:b_2_), representing the individual stages of compression on π–A isotherms, detailed in the text. The acronyms S40:S83, S65:S83, S80:S83 describe the evaluated seria of assessed surfactant mixtures according to the Table 5—Section 4.

The course of the π–A isotherm is determined both by structural factors, as well as by the forces acting between the molecules densely packed in the late stages of compression [39]. In our previous work, we described the model, which may elucidate and predict the values of HLB using elasticity modules and molecules surfaces [24]. However, we concentrated on another, more simplified approach to understand if there is linear coincidence between the direct parameters of π–A isotherms and an HLB value. Therefore, another set of surfactants was used. The selected surfactant systems were characterized by various basic HLB values (Table 2), and structures (Figure 1), different from those studied formerly by our team. The ratios of the slopes of the linear sections of the plots of the π–A isotherms, as well as the interceptions of the extrapolations of the sections, turned out to be the most promising (Figure 3 and Table 4).

The linear regression applied to the plot, which represented the relationship of the ratios of the slopes for HLBs, gave the highest determination coefficients for the systems with sorbitan monooleate and sorbitan sesquioleate (S80:S83, Table 4).

The lowest determination coefficient was definitely obtained for the seria of systems composed of sorbitan tristearate and sorbitan sesquioleate (S65:S83, Table 4). In the case of data obtained from the monolayers based on sorbitan monopalmitate and sorbitan sesquioleate (S40:S83, Table 4), the determination coefficients were on the intermediate level. Similar dependence was observed in the case of the interception ratios. However, the regression revealed that the most appropriate factor may be the slope ratio, which was 0.9468 for the S80:S83 system.

The high determination coefficient for the linear regression of the slope ratio and HLB in the case of the monolayers composed of monooleate derivatives (S80:S83) may be ascribed to the similarity in the components of sorbitan monooleate and sorbitan sesquioleate. In fact, sorbitan sesquioleate contains in its composition a fraction of sorbitan monooleate. Thus, the addition of the doping surfactant in this case alters non-extensively the monolayers’ structure. Another factor which may add to the high determination coefficient may be the close HLB value of both surfactants, which are 3.70 and 4.30, respectively. In the system with the lowest determination coefficient, the molecular structures were more different in terms of the number of carbon chains attached to the sorbitan core, as well as the HLB values which were definitively distant at 2.10 and 6.70, respectively. The intermediate situation was ascribed to the system S40:S83, where both the distance between the HLB values as well as the structural differences were less expressed—both surfactants included one carbon chain connected to the sorbitan, although with varied bonds, and the sorbitan monopalmitate was saturated and the sorbitan sesquioleate was non-saturated. The relationship between π-A isotherms and HLB may be more easily assessed in the mixtures of surfactants with similar structural patterns in the future. According to the studies of other authors, amphiphilic properties may influence the behavior of compressed monolayers [40,41,42,43]. Thus, the range of HLBs may be of high importance in this process, which should be further developed based on the presented results, and the data gathered in our former work [24] for dermatological and pharmaceutical applications.

The proposed HLB evaluation method can enable a quick comparison of some new surfactants evaluated in Langmuir balance during the development of innovative surfactants and give practical insight into the properties of newly proposed or evaluated surfactants and surfactant mixtures. An interesting question is also whether the parameters important for the evaluation of the surfactant structure can also serve as an indicator of their direct application value.

## 4. Materials and Methods

### 4.1. Materials

Span 40 (sorbitan monopalmitate), Span 65 (sorbitan tristearate), Span 80 (sorbitan monooleate), Span 83 (sorbitan sesquioleate), and all of purity > 99% were purchased from Merck, Warszawa, Poland. Deionized water with a conductivity below 2 µS/cm was used in the experiments. Solutions of surfactant mixtures for the monolayer preparations were prepared in chloroform (p.a. purity). Isopropyl alcohol (p.a. purity) was used to clean the Langmuir trough.

### 4.2. Composition of the Evaluated Mixtures of Surfactants

The assessed surfactants were dissolved in chloroform of p.a. purity. The stock solutions’ concentrations were within the range of 1 mg/mL. Mixtures of the required molar fractions were obtained by mixing the calculated volumes of stock solutions immediately before application onto the subphase. The volume of the applied surfactant solutions was set at 5 microliters. The measurements were performed at 25 +/− 1 °C. The composition of the resulting solutions are presented in Table 5. 

### 4.3. Surface Pressure Measurements

The π-A isotherms were recorded in a Kibron Micro Trough (Kibron, Helsinki, Finland). A measuring element in the form of a platinum rod was flame-cleaned with a gas burner. FilmwareX 4.0 was used to control the experiment and to record the results. The calibration of the device is based on the tabulated value of the subphase surface tension and the standard mass of a detector. The chloroform (Merck, Warszawa, Poland) solution of the tested surfactants and their mixtures were placed 5 cm apart in distance and 5 cm away from one of the barriers. After completion of the evaporation of the solvent, which followed the 15 min of monolayer conditioning, the measurements started. Externally applied transparent coating protected the subphase against solid molecules from the air. The barrier speed was 14.5 mm/min.

### 4.4. Calculation of the Selected Parameters of the Evaluated π-A Isotherms

On the obtained plots of π-A isotherms, the segments representing the first and second stages of compression were selected according to the linear sections of the plots. The evaluated sets of data were limited to the segments with high Pearson’s coefficients. The respective slopes and interceptions with the y-axis were calculated due to the linear equation y=ax+b, obtained from linear regression calculations.

### 4.5. Calculation of HLB of the Assessed Surfactants Mixtures

The HLB values were calculated according to the Griffin concept of the balance [14]. The fractional HLB of the applied surfactants were treated as additive values. The following formula was used, where the index of HLB by Griffin of the selected surfactant (${HLB}_{i}$) is multiplied by his weight fraction in the system ($f_{i}$):

(3) $$HLB=\sum {HLB}_{i}\times f_{i}$$

## 5. Conclusions

We identified some possible patterns between the calculated values of HLB for the evaluated surfactants systems and the parameters of π–A isotherms obtained from monolayer compression studies. The selection of the ratio of the slope in the first stage of monolayer compression to the second stage of the layer compression enabled a comparison of the considered value of HLB to the recorded π–A isotherms. The relation of the calculated HLB values and the registered intercepts ratios was most remarkable in the case of the systems containing sorbitan monooleate and sorbitan sesquioleate. The relationship of the slope ratio to HLB was linear, with a coefficient of determination of 0.95, while the linearization of the relationship of the intercept ratio to HLB gave a lower coefficient of determination of 0.71. In the systems composed of spans 65 and 80, as well as the systems with spans 40 and 83, the relation was inexpressive. In summary, it is possible to reveal functional relationships that may enable the recovery of HLB from Langmuir monolayer studies, i.e., the slope and interception of the π-A isotherms. However, further studies with Langmuir balance are required to excavate more data on the relations between π-A isotherms and HLB value, including a visualization of the monolayers in Brewster angle microscopy or scanning electron microscopy. 

## Figures and Tables

**Table 1:** Selected properties of evaluated sorbitan esters.

AN	CSN	PG	NPG	NCC	NC	DB	M
S40	Sorbitan monopalmitate	1,4-sorbitan	Palmitic acid	16	1	N	402.57
S65	Sorbitan tristearate	1,4-sorbitan	Stearic acid	18	3	N	963.54
S80	Sorbitan monooleate	1,4-sorbitan	Oleic acid	18	1	Y	428.6
S83	Sorbitan sesquioleate *	1,4-sorbitan	Oleic acid	18	1.5	Y	1175.7

AN—abbreviated name, CSN—common synonym name, PG—polar group, NPG—nonpolar group, NCC—number of chain carbons, including carboxyl group, NC—number of chains per sorbitol molecule, DB—double bond, M—molar mass, and * indicates a mixture of oleic acid esters and hexitol anhydride.

**Table 2:** Linear equations y = ax + b for the first (a_1_, b_1_) and second (a_2_, b_2_) observed stages on π–A isotherms of the assessed systems. The slope was calculated as µN/mm^3^, and reflects the ratio π:A, whereas the intercept has a unit of mN/m, and reflects the intercept of the analyzed plot section with the y-axis. The composition of surfactants mixtures is presented in Table 5. The R2 is the determination coefficient.

Surfactants Mixture	Slope (a_1_)	Intercept (b_1_)	x_1_ Intercept for y = 0	R²	Slope (a_2_)	Intercept (b_2_)	x_2_ Intercept for y = 0	R²	Slopes Ratio a_1_:a_2_	Intercepts Ratio b_1_:b_2_
S4000A	−9.50 × 10^−3^	54.12	5.70 × 10^3^	0.9999	−2.63 × 10^−2^	84.24	3.20 × 10^3^	0.9986	2.77	0.64
S4083B	−9.90 × 10^−3^	62.97	6.36 × 10^3^	0.999	−1.36 × 10^−2^	66.87	4.92 × 10^3^	0.9834	1.37	0.94
S4083C	−1.13 × 10^−2^	71.21	6.30 × 10^3^	0.9975	−1.72 × 10^−2^	79.99	4.65 × 10^3^	0.9852	1.52	0.89
S4083D	−5.70 × 10^−3^	46.14	8.09 × 10^3^	0.9995	−9.50 × 10^−3^	57.02	6.00 × 10^3^	0.9996	1.67	0.81
S6500A	−2.16 × 10^−2^	127.42	5.90 × 10^3^	0.9947	−4.00 × 10^−3^	72.17	1.80 × 10^4^	0.9731	0.19	1.77
S6583B	−2.94 × 10^−2^	85.031	2.89 × 10^3^	0.9914	−5.19 × 10^−2^	108.58	2.09 × 10^3^	0.9932	1.77	0.78
S6583C	−7.80 × 10^−3^	56.482	7.24 × 10^3^	0.9994	−3.12 × 10^−2^	79.535	2.55 × 10^3^	0.9877	4.00	0.71
S6583D	−5.90 × 10^−3^	56.184	9.52 × 10^3^	0.9985	−5.00 × 10^−3^	51.78	1.04 × 10^4^	0.9998	0.85	1.09
S8000A	−4.10 × 10^−3^	43.63	1.06 × 10^4^	0.9994	−6.60 × 10^−3^	50.30	7.62 × 10^3^	0.9996	1.61	0.87
S8083B	−4.40 × 10^−3^	42.94	9.76 × 10^3^	0.9995	−5.90 × 10^−3^	49.25	8.35 × 10^3^	0.9992	1.34	0.87
S8083C	−4.20 × 10^−3^	50.56	1.20 × 10^4^	0.9981	−4.20 × 10^−3^	50.58	1.20 × 10^4^	0.9932	1.00	1.00
S8083D	−4.10 × 10^−3^	49.64	1.21 × 10^4^	0.9995	−2.40 × 10^−3^	46.68	1.95 × 10^4^	0.9964	0.59	1.06
S0083E	−1.33 × 10^−2^	71.68	5.39 × 10^3^	0.9992	−4.00 × 10^−3^	45.45	1.14 × 10^4^	0.9975	0.30	1.58

The acronyms and composition of evaluated systems are given in Table 5 in Section 4.

**Table 3:** Calculated HLB values of evaluated systems; the fractional HLB of the applied surfactants were treated as additive values. The basic data were established from the literature [29,30,31].

Monolayer type	S40:S83	HLB	S65:S83	HLB	S80:S83	HLB
Evaluated samples	S4000A	6.70	S6500A	2.10	S8000A	4.30
	S4083B	5.62	S6583B	2.61	S8083B	4.06
	S4083C	5.11	S6583C	3.01	S8083C	3.98
	S4083D	4.25	S6583D	3.39	S8083D	3.86
	S0083E *	3.70	S0083E *	3.70	S0083E *	3.70

* The HLBs are identical but were presented separately for clear evaluation; the composition of the evaluated mixtures of the surface-active agents are given in molar fractions.

**Table 4:** Linear equation, y = ax + b, for the following relations: slope ratios v. HLB, and intercept ratios v. HLB, with values from Table 2; the plots are depicted in Figure 3.

Type of Composition	Slopes Ratio a_1_:a_2_ v. HLB		Intercepts Ratio b_1_:b_2_ v. HLB	
	Equation	Determination Coefficient	Equation	Determination Coefficient
S40:S83	y = 0.6349x − 1.6969	0.7204	y = −0.227x + 2.1244	0.5585
S65:S83	y = 0.0004x + 1.4185	3.0 × 10^−8^	y = −0.0848x + 1.4352	0.0129
S80:S83	y = 2.3215x − 8.272	0.9468	y = −1.1015x + 5.4595	0.7118

**Table 5:** Composition of evaluated surfactants systems.

Monolayer Type	S40:S83		Monolayer Type	S65:S83		Monolayer Type	S80:S83	
Components	Molar Fraction		Components	Molar Fraction		Components	Molar Fraction	
	S40	S83		S65	S83		S80	S83
S4000A	1.00	0.00	S6500A	1.00	0.00	S8000A	1.00	0.00
S4083B	0.84	0.16	S6583B	0.73	0.28	S8083B	0.80	0.20
S4083C	0.72	0.28	S6583C	0.48	0.52	S8083C	0.71	0.29
S4083D	0.39	0.61	S6583D	0.23	0.77	S8083D	0.50	0.50
S0083E *	0.00	1.00	S0083E *	0.00	1.00	S0083E *	0.00	1.00

* These compositions are identical but were presented separately for clear evaluations; the composition of the evaluated mixtures of surface-active agents are given in molar fractions.

## Data Availability

The original contributions presented in this study are included in the article. Further inquiries can be directed to the corresponding author.
